# Spatial Mechano‐Signaling Regulation of GTPases through Non‐Degradative Ubiquitination

**DOI:** 10.1002/advs.202303367

**Published:** 2023-11-09

**Authors:** Raj N. Sewduth, Paolo Carai, Tonci Ivanisevic, Mingzhen Zhang, Hyunbum Jang, Benoit Lechat, Delphi Van Haver, Francis Impens, Ruth Nussinov, Elizabeth Jones, Anna Sablina

**Affiliations:** ^1^ VIB‐KU Leuven Center for Cancer Biology VIB Leuven 3000 Belgium; ^2^ Department of Oncology KU Leuven Herestraat 49 Leuven 3000 Belgium; ^3^ Department of Cardiovascular Sciences Centre for Molecular and Vascular Biology KU Leuven Herestraat 49 Leuven 3000 Belgium; ^4^ Computational Structural Biology Section Frederick National Laboratory for Cancer Research in the Laboratory of Cancer ImmunoMetabolism National Cancer Institute Frederick MD 21702 USA; ^5^ VIB‐UGent Center for Medical Biotechnology Technologiepark‐Zwijnaarde 75 Ghent 9052 Belgium; ^6^ Department of Biomolecular Medicine Ghent University Technologiepark‐Zwijnaarde 75 Ghent 9052 Belgium; ^7^ VIB Proteomics Core Technologiepark‐Zwijnaarde 75 Ghent 9052 Belgium; ^8^ Department of Human Molecular Genetics and Biochemistry Sackler School of Medicine Tel Aviv University Tel Aviv 69978 Israel; ^9^ Department of Cardiology CARIM School for Cardiovascular Diseases Maastricht University Universiteitssingel 50 Maastricht 6229 ER The Netherlands

**Keywords:** endothelial cells, GTPase, mechanobiology, spatial OMICs, ubiquitin system

## Abstract

Blood flow produces shear stress exerted on the endothelial layer of the vessels. Spatial characterization of the endothelial proteome is required to uncover the mechanisms of endothelial activation by shear stress, as blood flow varies in the vasculature. An integrative ubiquitinome and proteome analysis of shear‐stressed endothelial cells demonstrated that the non‐degradative ubiquitination of several GTPases is regulated by mechano‐signaling. Spatial analysis reveals increased ubiquitination of the small GTPase RAP1 in the descending aorta, a region exposed to laminar shear stress. The ubiquitin ligase WWP2 is identified as a novel regulator of RAP1 ubiquitination during shear stress response. Non‐degradative ubiquitination fine‐tunes the function of GTPases by modifying their interacting network. Specifically, WWP2‐mediated RAP1 ubiquitination at lysine 31 switches the balance from the RAP1/ Talin 1 (TLN1) toward RAP1/ Afadin (AFDN) or RAP1/ RAS Interacting Protein 1 (RASIP1) complex formation, which is essential to suppress shear stress‐induced reactive oxygen species (ROS) production and maintain endothelial barrier integrity. Increased ROS production in endothelial cells in the descending aorta of endothelial‐specific *Wwp2*‐knockout mice leads to increased levels of oxidized lipids and inflammation. These results highlight the importance of the spatially regulated non‐degradative ubiquitination of GTPases in endothelial mechano‐activation.

## Graphical abstract

An integrative ubiquitinome and proteome analysis demonstrates that the non‐degradative ubiquitination of several GTPases is regulated by mechano‐signaling. Spatial analysis reveals RAP1 ubiquitination specifically in the descending aorta, a region exposed to laminar shear stress. The ubiquitin ligase WWP2‐mediated RAP1 ubiquitination at lysine 31 switches the balance toward the RAP1/ RASIP1 complex formation, which is essential for maintaining endothelial barrier function.

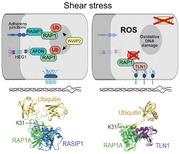

## 1 Introduction

Shear stress, which is exerted on the lumen of the blood vessel and directly activates endothelial cells (EC), is dysregulated in several cardiovascular diseases, including atherosclerosis, cardiac hypertension, cardiac failure, and stroke, all which are leading causes of death worldwide.^[^ ^1^ ^]^ Regions of the arterial circulation are exposed to uniform physiologic laminar shear stress (LSS) that confers a vasculoprotective effect, whereas regions of low or recirculating shear stress near arterial branches and bifurcations are more prone to vascular disease development,^[^ ^2^, ^3^ ^]^ highlighting the need for spatial approaches for a better understanding of mechanotransduction mechanisms.^[^ ^4^, ^5^ ^]^ Vascular lesions occur not only in vulnerable regions of the circulation exposed to pathological shear stress but also in case of disruption of the normal LSS response in endothelial cells.

As shear stress coordinates multiple interconnected intracellular processes by controlling the expression and post‐translational modification of thousands of proteins,^[^ ^6^ ^]^ unbiased systems biology‐based approaches are essential to fully elucidate the interaction between mechanical forces and vascular physiology. Although multiple studies explored gene expression regulation in endothelial cells in response to shear stress in a low throughput manner, the landscape of post‐translational alterations during mechanotransduction remains incompletely understood.

Recent studies highlighted the role of the ubiquitin system in endothelial homeostasis and associated cardiovascular pathologies.^[^ ^7^, ^8^, ^9^ ^]^ Ubiquitination is an intracellular process coordinated by three enzymes, an E1 ubiquitin‐activating enzyme, an E2 ubiquitin‐conjugating enzyme, and an E3 ubiquitin ligase, that gives specificity to the process.^[^ ^10^ ^]^ Ubiquitin can be conjugated to a protein substrate, either as a single ubiquitin molecule (mono‐ubiquitination) or as several independent ubiquitin molecules (multiple mono‐ubiquitination).^[^ ^11^, ^12^ ^]^ Ubiquitination is a reversible and dynamically regulated post‐translational modification of proteins, regulated notably by deubiquitinating enzymes.^[^ ^13^ ^]^ There is increasing evidence that ubiquitination not only serves to target the substrate for proteasomal degradation, but also controls cellular functions in many ways, including regulation of protein‐protein interactions, vesicular trafficking, and subcellular localization of signaling proteins.^[^ ^14^ ^]^

By integrating the results of proteome and ubiquitome analyses, we revealed shear stress‐driven perturbations of the ubiquitin system in endothelial cells. We found that non‐degradative ubiquitination fine‐tunes the function of GTPases to maintain endothelial homeostasis. We determined the ubiquitination profiles of RAP1 in aortic vascular beds, exposed to differential shear stress. The dysregulation of WWP2‐mediated RAP1 ubiquitination leads to increased production of reactive oxygen species (ROS) and oxidative DNA damage in the endothelial layer of the descending aorta, a region exposed to laminar shear stress. Increased ROS production induced by dysregulation of RAP1 ubiquitination affected the endothelial barrier integrity. Our study provides a global view and a mechanistic explanation for the contribution of the ubiquitin system in the shear stress response in endothelial cells, highlighting a spatial regulation of RAP1 ubiquitination to dynamically drive cellular plasticity.

## 2 Results and Discussion

### 2.1 Laminar Shear Stress‐Induced Perturbations of the Ubiquitin Landscape of Endothelial Cells

To identify shear stress‐driven perturbations in endothelial cells, we assessed global proteomics and ubiquitome alterations triggered by shear stress. Because it was not feasible to get a sufficient amount of proteins to perform the ubiquitinome analysis in endothelial cells subjected to shear stress with a parallel plate flow chamber, we mimicked shear stress using the activator of PIEZO1 channels YODA1.^[^ ^15^, ^16^, ^17^ ^]^ PIEZO1 channels are key sensors of shear stress that promote activation of shear stress‐related signaling.^[^ ^18^, ^19^ ^]^ Concordantly, YODA1 treatment of Human Umbilical Vein Endothelial Cells (HUVECs) led to increased phosphorylation of the endothelial nitric oxide synthase eNOS (NOS III) and Mitogen‐activated protein kinase 1/2 (MEK1/2), both known to be induced by shear stress (Figure S1a, Supporting Information). Further analysis of calcium signaling monitored by the expression levels of the regulator of calcineurin 1 (RCAN1) revealed similar patterns in YODA1‐treated or LSS‐exposed ECs (Figure S1b, Supporting Information). Both LSS as well as YODA1 treatment also transiently induced ROS levels to a comparable level (Figure S1c, Supporting Information).

**Figure 1:**
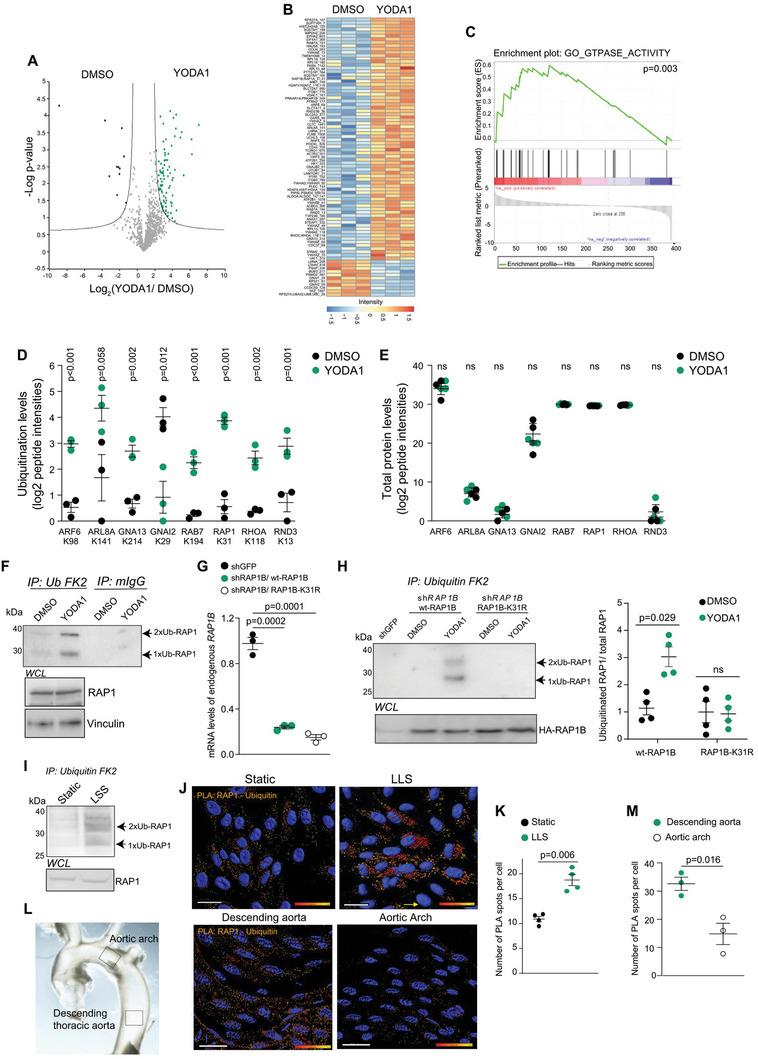
Shear stress leads to non‐degradative ubiquitination of GTPases. a,b) A volcano plot and a heatmap showing YODA1‐induced ubiquitinome alterations in HUVECs. Three independent pools of HUVECs were treated with YODA1 (48 h, 3 µm). a) The cutoff of *p*  =  0.05 and the two‐fold change is marked by grey lines. b) The scale shows Z‐scored site intensity values. c) Gene set enrichment analysis (GSEA) of YODA1‐mediated ubiquitome alterations in HUVECs. d,e) Ubiquitination levels (d) and protein abundance (e) of the indicated GTPases in mock or YODA1‐treated HUVECs detected by MS. Data is shown as mean ± SEM. *N* = 3. *p*‐values, the Welch's *t*‐test. f) RAP1 ubiquitination in mock or YODA1‐treated (48 h, 3 µm) HUVECs. Ubiquitinated proteins were immunoprecipitated using anti‐ubiquitin FK2 beads. g,h) wt‐RAP1B and RAP1B‐K31R ubiquitination in mock or YODA1‐treated (48 h, 3 µm) HUVECs. HUVECs were infected with sh*GFP* or dual expression plasmids expressing sh*RAP1B* and HA‐tagged wt‐RAP1B or RAP1B‐K31R. g) RT‐qPCR analysis of RAP1B expression in the indicated HUVECs. Data is shown as mean ± SEM. *N* = 3. *p*‐values, the Wilcoxon Mann–Whitney test. h) Ubiquitinated proteins were immunoprecipitated using anti‐ubiquitin FK2 beads. Quantification of RAP1 ubiquitination levels is shown as mean ± SEM. *N* = 4. *p*‐values, the Wilcoxon Mann–Whitney test. i) RAP1 ubiquitination levels in static or shear stress‐exposed HUVECs. HUVECs were subjected to LSS, 6 dyn cm^−2^, 24 h. j,k) RAP1 ubiquitination in static or shear stress‐activated HUVECs detected by PLA using a pair of anti‐RAP1 and anti‐ubiquitin antibodies. HUVECs were subjected to LSS, 6 dyn cm^−2^, 24 h. j) Scale bar 20 µm. k) The number of PLA spots per nucleus is shown as mean ± SEM. *N* = 3. *p*‐value, the Wilcoxon Mann–Whitney test. l,m) RAP1 ubiquitination in mouse aortic arch and descending aorta was detected by PLA using a pair of anti‐RAP1 and anti‐ubiquitin antibodies. l) Scale bar 20 µm µm. m) The number of PLA spots per nucleus is shown as mean ± SEM. N = 3 each. *p*‐values, the Wilcoxon Mann–Whitney test. WCL, whole cell lysate.

As YODA1 treatment recapitulated several features of physiological LSS, we then interrogated ubiquitome and proteome alterations induced by shear stress in three independent pools of mock or YODA1‐treated HUVEC cells. Out of the 1411 ubiquitination events quantified by the mass‐spectrometry (MS)‐based ubiquitome analysis, 89 events showed statistically significant changes in ubiquitination in YODA1‐treated HUVECs (**Figure** **1**a,b). Among differentially ubiquitinated proteins, we observed a significant enrichment of GTPase proteins (Figure 1c), including heteromeric G‐proteins and small GTPases (Figure 1d). On the other hand, GTPase protein expression levels were not affected by YODA1 treatment (Figure 1e), suggesting that non‐degradative ubiquitination represents a novel mechanism of regulation for GTPase proteins in response to mechano‐signaling activation.

Non‐degradative ubiquitination of GTPases identified in our OMIC analysis has been proposed to fine‐tune their functions by altering their interaction network.^[^ ^20^ ^]^ For example, the ubiquitination of the small GTPase RAB7 at K194 (Figure 1d) has been shown to induce its binding to vacuolar protein sorting 35 (VPS35), thereby maintaining spatiotemporal order in the endosomal system.^[^ ^21^ ^]^ The ubiquitination of ADP‐Ribosylation Factor 6 (ARF6) at K69 (Figure 1d), has been suggested to reduce its affinity with the downstream effector multidomain ARF GAP protein (AMAP1), potentially affecting endothelial cell migration and tubulogenesis.^[^ ^22^ ^]^ In contrast to these examples, the role of shear stress‐induced ubiquitination on the function of the RAP1A and RAP1B GTPases (Figure 1d) remains unexplored, even though these two highly homologous RAP1 isoforms are key regulators of endothelial function.^[^ ^23^, ^24^ ^]^ Therefore, we focused here on exploring the contribution of RAP1 ubiquitination in maintaining endothelial function during shear stress response.

### 2.2 Laminar Shear Stress Induces Non‐Degradative Ubiquitination of RAP1 at K31

We validated increased RAP1 ubiquitination in response to YODA1 by pulling down ubiquitinated proteins from HUVECs using an anti‐ubiquitin FK2 antibody. YODA1 treatment led to the accumulation of mono‐ and di‐ubiquitinated forms of RAP1 (Figure 1f). We then generated dual expression constructs that concurrently suppressed the expression of endogenous *RAP1B* and overexpressed either wild‐type (wt)‐RAP1B or ubiquitination‐deficient RAP1B‐K31R mutant (Figure 1g,h). In line with the MS results, YODA1‐treated HUVECs showed increased ubiquitination of wt‐RAP1, whereas K31R mutation abrogated YODA1‐mediated ubiquitination of RAP1 (Figure 1g). We also did not observe a decrease in RAP1 protein expression in response to YODA1 (Figure 1f,h). These results confirm that YODA1 induces non‐degradative ubiquitination of RAP1 at K31.

We also observed a clear accumulation of mono‐ and di‐ubiquitinated forms of RAP1 in HUVECs exposed to physiological LSS, whereas RAP1 protein expression was not changed (Figure 1i). The proximity ligation assay (PLA) with a pair of anti‐ubiquitin and anti‐RAP1 antibodies confirmed that LSS led to increased levels of RAP1 ubiquitination (Figure 1j,k). To validate these results in vivo and assess the spatial localization of RAP1 ubiquitination, we performed an *en* *face* PLA staining of the mouse aorta vascular layer. The aortic arch presents areas of atheroprone shear stress patterns, whereas the descending aorta is exposed to higher laminar stress.^[^ ^25^, ^26^, ^27^, ^28^, ^29^ ^]^ We detected higher levels of RAP1 ubiquitination in the endothelial cells of the descending aorta when compared to the aortic arch (Figure 1l,m). Given that K31 is the major site of RAP1B ubiquitination in mice (Figure S1d, Supporting Information), our data indicate that laminar shear stress triggers non‐degradative ubiquitination of RAP1 at K31 specifically in the descending aorta.

### 2.3 The Ubiquitin Ligase WWP2 Mediates RAP1 Ubiquitination in Response to Shear Stress

**Figure 2:**
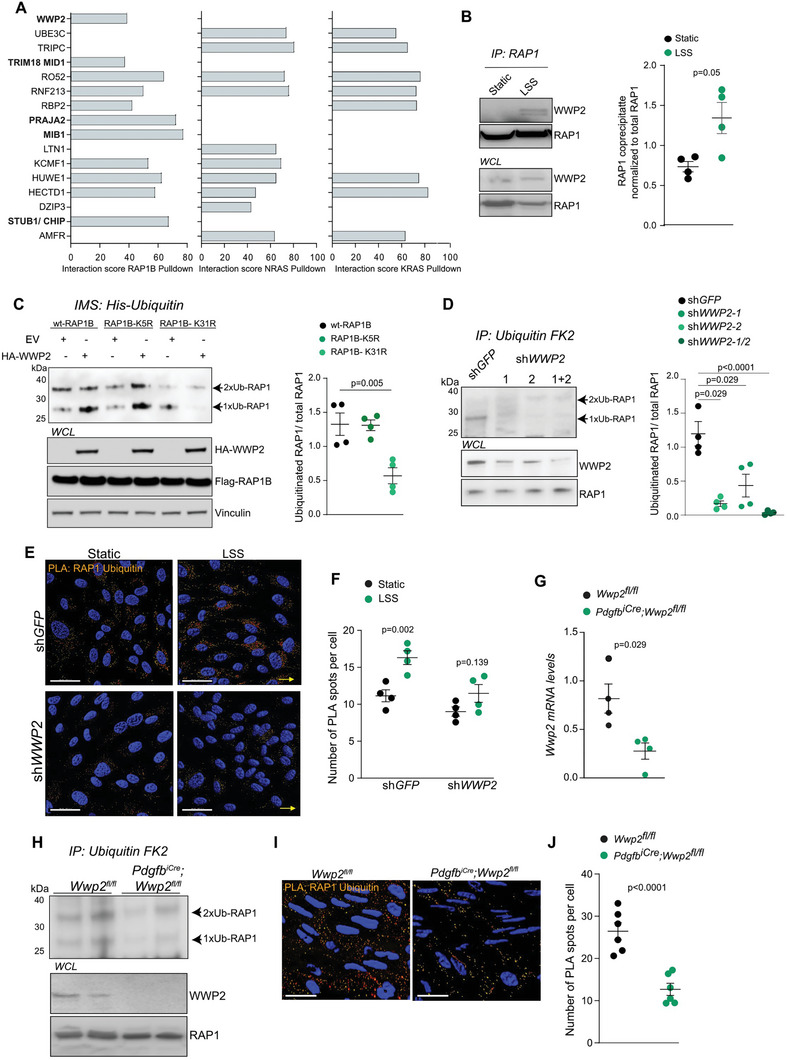
The ubiquitin ligase WWP2 promotes shear stress‐induced ubiquitination of RAP1 at K31. a) MS analysis of tandem purified Flag‐tagged RAP1B, NRAS, or KRAS immunoprecipitates. Flag‐tagged GTPases were overexpressed in HEK293T cells. The average interaction score is shown for all identified E3 ubiquitin ligases. E3 ubiquitin ligases specific to RAP1B immunoprecipitate are shown in bold. b) The interaction between WWP2 and RAP1 in static or shear stress‐activated HUVECs. HUVECs were subjected to LSS, 6 dyn cm^−2^, 24 h. Endogenous RAP1 proteins were immunoprecipitated using agarose conjugated beads followed by immunoblotting with anti‐WWP2 or anti‐RAP1 antibodies. Quantification of coprecipitate of RAP1 is shown is shown as mean ± SEM. *N* = 4. *p*‐values, the Wilcoxon Mann–Whitney test. c) Ubiquitination of RAP1B‐WT or the indicated RAP1B mutants in HEK293T expressing an empty vector (EV) or HA‐tagged WWP2 together with 6xHis‐tagged ubiquitin Flag‐tagged RAP1B constructs. Ubiquitinated RAP1B was purified by Co^2+^ metal affinity chromatography and detected by anti‐Flag antibodies. Quantification of RAP1 ubiquitination levels is shown as mean±SEM. *N* = 4. *p*‐values, one‐way ANOVA with Bonferroni post‐tests. d) RAP1 ubiquitination levels in HUVECs expressing sh*GFP* or sh*WWP2*. Ubiquitinated proteins were immunoprecipitated using anti‐ubiquitin FK2 beads. Quantification of RAP1 ubiquitination levels is shown as mean±SEM. *N* = 4. *p*‐values, one‐way ANOVA with Bonferroni post‐tests. e,f) RAP1 ubiquitination in static or shear stress‐activated HUVECs expressing sh*GFP* or sh*WWP2* was detected by PLA using a pair of anti‐RAP1 and anti‐ubiquitin antibodies. HUVECs were subjected to LSS, 6 dyn cm^−2^, 24 h. e) Scale bar 20 µm. f) The number of PLA spots per cell is shown as mean±SEM. N = 4. *p*‐value, the Wilcoxon Mann–Whitney test. g) *Wwp2* expression in sorted endothelial cells isolated from *Wwp2* ^*fl/fl*^ or *Pdgfb* ^*iCre*^; *Wwp* *2* ^*fl/fl*^ mice determined by RT‐qPCR. *N* = 4 per group. *p*‐value, the Wilcoxon Mann–Whitney test. h) RAP1 ubiquitination in sorted endothelial cells isolated from *Wwp2* ^*fl/fl*^ or *Pdgfb* ^*iCre*^; *Wwp2* ^*fl/fl*^ mice. Ubiquitinated proteins were immunoprecipitated using anti‐ubiquitin FK2 beads. i,j) RAP1 ubiquitination in descending aortas of *Wwp2* ^*fl/fl*^ or *Pdgfb^iCre^*; *Wwp2^fl^^/^^fl^* mice at age 15–16 weeks, injected with tamoxifen at weaning. RAP1 ubiquitination was determined by PLA using a pair of anti‐RAP1 and anti‐ubiquitin antibodies. i) Scale bar 20 µm. j) The number of PLA spots per nucleus is shown as mean; *N* = 6 per group. *p*‐value, the Wilcoxon Mann–Whitney test. WCL, whole cell lysate.

Given that ubiquitin enzymes are promising drug targets to modulate endothelial homeostasis, we screened for the ubiquitin machinery controlling RAP1 ubiquitination.^[^ ^30^ ^]^ Using tandem affinity purification of RAP1B followed by MS, we identified several previously described RAP1 interactors in the RAP1 immunoprecipitate (Figure S2a, Supporting Information). We also identified several ubiquitin ligases, such as WW Domain Containing E3 Ubiquitin Protein Ligase 2 (WWP2), MIB1, and CHIP, as putative and specific RAP1B interactors (**Figure** **2**a). On the other hand, the Ubibrowser analysis ^[^ ^31^ ^]^ of RAP1A and RAP1B predicts the highest ubiquitin ligase‐ substrate interaction scores for the HECT (Homologous to E6AP C‐Terminus) E3 ligases of the NEDD4 family, including WWP2 (Figure S2b, Supporting Information). The ubiquitin ligase WWP2 showed high scores in both datasets (Figure 2a; Figure S2b, Supporting Information). Moreover, recent studies strongly indicated the role of WWP2 in cardiovascular function,^[^ ^32^ ^]^ whereas its contribution to vascular homeostasis is still unclear,^[^ ^33^ ^]^ as no endothelial substrates have been identified yet.

We confirmed the interaction between RAP1B and WWP2 in a set of reciprocal immunoprecipitations. HA‐tagged RAP1B coimmunoprecipitated (co‐IPed) with Flag‐tagged WWP2, whereas HA‐tagged WWP2 co‐IPed with Flag‐tagged RAP1B (Figure S2c,d). We also detected the interaction between endogenous RAP1 and WWP2 in HUVECs exposed to LSS (Figure 2b), suggesting that WWP2 could mediate the ubiquitination of RAP1 at K31 specifically in response to LSS. In line with this hypothesis, WWP2 overexpression induced the ubiquitination of either wt‐RAP1B or RAP1B‐K5R but did not affect the ubiquitination of the RAP1B‐K31R mutant (Figure 2c). We also observed that the overexpression of wt‐WWP2, but not the catalytically inactive WWP2A‐C838A mutant,^[^ ^34^ ^]^ led to increased levels of RAP1B ubiquitination (Figure S2e, Supporting Information). In contrast, WWP2 suppression led to a decrease in the levels of RAP1B ubiquitination in HEK293T cells (Figure S2f,g, Supporting Information). A decreased ubiquitination of endogenous RAP1 was also observed in HUVECs expressing sh*WWP2* (Figure 2d). The PLA using a pair of RAP1‐ and ubiquitin‐specific antibodies further demonstrated that WWP2 suppression abolished LSS‐induced ubiquitination of RAP1 in HUVECs (Figure 2e,f), indicating that WWP2 triggers RAP1 ubiquitination in shear stressed endothelial cells.

To characterize the role of WWP2 in the regulation of RAP1 ubiquitination in vivo, we generated an endothelial‐specific platelet derived growth factor subunit B (*Pdgfb)‐Cre* ^*ERT2 (iCre)*^ knockout mouse for *Wwp2*. Endothelial deletion of *Wwp2* at E8.5 led to early embryonic lethality caused by growth arrest, hydrops, or hemorrhages by E13.5 (Figure S3a,b, Supporting Information), indicating the vital function of WWP2 in cardiovascular development. Therefore, we induced the endothelial deletion of *Wwp2* in *Pdgfb‐Cre* ^*ERT2*^, *Wwp2* ^*fl* */* *fl*^ animals at the age of 3 weeks (Figure 2g). Sorted endothelial from *Pdgfb‐Cre* ^*ERT2*^, *Wwp2* ^*fl* */* *fl*^ mice showed a decreased RAP1 ubiquitination (Figure 2h). Moreover, *en face* staining of the descending aortic region revealed lower levels of RAP1 ubiquitination in the aorta of the *Wwp2* knockout mice (Figure 2i,j). Taken together, these results indicate that WWP2 regulates RAP1 ubiquitination in endothelial cells of the aorta regions exposed to LSS.

### 2.4 WWP2‐Mediated RAP1 Ubiquitination Determines its Choice of Downstream Effectors

We next evaluated how the ubiquitination of RAP1 at K31 affects its function. The MS‐based analysis of Flag‐tagged RAP1B‐G12V showed that the constitutively active mutant was also ubiquitinated at K31 (Figure S4a, Supporting Information). Furthermore, mutation of K31 to arginine dramatically decreased levels of ubiquitination wt‐RAP1B and the RAP1B‐G12V mutant, confirming that K31 is the major site of ubiquitination of either GDP‐ or GTP‐bound RAP1 (Figure S4b, Supporting Information).

As shear stress RAP1 function^[^ ^24^ ^]^(Figure S4c, Supporting Information), we hypothesized that RAP1B ubiquitination might induce its activity. However, both wt‐RAP1B and the ubiquitination‐deficient RAP1B‐K31R mutant showed similar activity levels (Figure S4d, Supporting Information). Additionally, we observed similar levels of ubiquitination of wt‐RAP1B and the RAP1B‐G12V mutant, suggesting that RAP1 undergoes ubiquitination independently of its GTP‐ or GDP‐bound status. Altogether, these results indicate that ubiquitination and activation of RAP1 are not coupled events.

**Figure 3:**
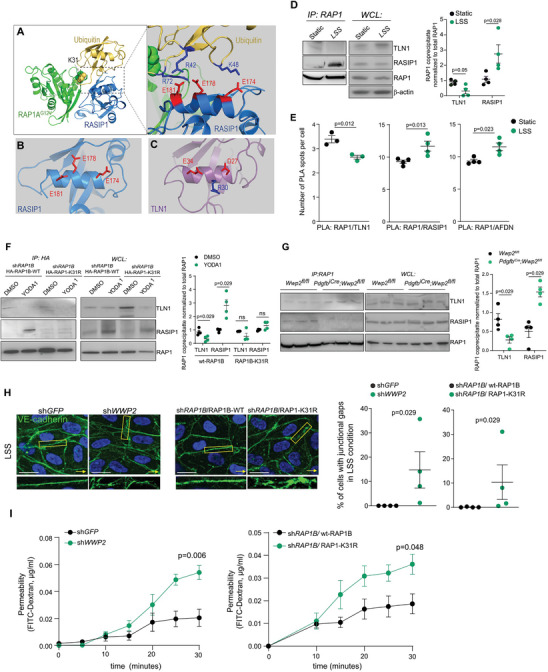
Shear stress‐induced ubiquitination of RAP1 modifies its affinity for downstream effectors. a,b,c) In silico modeling of the interaction between ubiquitinated RAP1 and its downstream effectors RASIP1 and TLN1. Ubiquitin conjugated to K31 of RAP1‐G12V forms multiple salt bridges with RASIP1. b,c) In silico modeling shows the different surface residues in RASIP1 (b) and TLN1 (c) accommodating ubiquitin conjugated to RAP1 at K31. d,e) The interaction between RAP1 and RASIP1 or TLN1 in HUVECs exposed to LSS, 6 dyn cm^−2^, 24 h. d) RAP1 was immunoprecipitated from static and shear stress‐activated HUVECs followed by immunoblotting with antibodies specific to RAP1, TLN1, and RASIP1. Quantification of coprecipitate of RAP1 is shown as mean ± SEM. *N* = 4. *p*‐values, the Wilcoxon Mann–Whitney test. e) The PLA using anti‐RAP1 and anti‐TLN1, anti‐RASIP1, or anti‐AFDN antibodies. The number of PLA spots per nucleus is shown as mean ± SEM; *N* = 3–4. *p*‐values, the Wilcoxon Mann–Whitney test. f) The interaction between wt‐RAP1B or RAP1B‐K31R and RASIP1 or TLN1 in mock HUVECs or HUVECs treated with YODA1 (3 µm, 48 h). HA‐tagged RAP1 was immunoprecipitated using anti‐HA agarose conjugated beads followed by immunoblotting with antibodies specific to HA, TLN1, and RASIP1. Quantification of coprecipitate of RAP1 is shown as mean ± SEM. *N* = 4. *p*‐values, the Wilcoxon Mann–Whitney test. g) The interaction between RAP1 and RASIP1 or TLN1 in sorted endothelial cells isolated from *Wwp2* ^*fl/fl*^ or *Pdgfb* ^i*Cre*^; *Wwp2* ^*fl/fl*^ mice. RAP1 was immunoprecipitated with anti‐RAP1 antibody followed by immunoblotting with anti‐RAP1, TLN1, or RASIP1 antibodies. Quantification of coprecipitate of RAP1 is shown as mean ± SEM. *N* = 4. *p*‐values, the Wilcoxon Mann–Whitney test. h) VE‐cadherin immunostaining of shear stress‐activated HUVECs expressing the indicated constructs. HUVECs were exposed to LSS, 6 dyn cm^−2^, 24 h. Scale bar, 10 µm.Yellow arrows show the direction of flow. The percentage of cells with junctional gaps is shown as mean ± SEM; *N* = 4. *p*‐values, the Wilcoxon Mann–Whitney test. i) FITC‐Dextran permeability of the monolayer of HUVECs expressing the indicated constructs. Data is shown as mean ± SEM. *N* = 5. *p*‐values, the two‐way ANOVA.

Shear stress‐induced RAP1 ubiquitination at K31, which is located within the effector‐binding domain of RAP1 (Figure 1d), might also tune its binding toward specific downstream effectors. RAP1 is a key regulator of cell plasticity that controls focal adhesions by binding to Rap Associating With DIL Domain (RADIL), RAP1‐GTP‐interacting adaptor molecule (RIAM), Talin 1 (TLN1),^[^ ^35^, ^36^, ^37^, ^38^ ^]^ and the stability of adherent junctions through Afadin (AFDN), RAS Interacting Protein 1 (RASIP),^[^ ^39^, ^40^ ^]^ and KRIT1.^[^ ^41^ ^]^ To assess the impact of RAP1 ubiquitination at K31 on its ability to bind its effectors, we performed in silico modeling using the structures available for the RAP1A‐G12V/ RASIP1 and RAP1A‐G12V/ TLN1 complexes. We found that the initial RAP1A‐G12V/ RASIP1 interaction surface was maintained upon ubiquitination, and the key contact residues at the RAP1A‐G12V/ RASIP1 interface remained stable (Figure S5a–c, Supporting Information). Moreover, ubiquitin conjugated to RAP1A at K31 (model 2) forms stable interactions with RASIP1, featuring multiple interfacial salt bridges (**Figure** **3**a,b; Figure S5d, Supporting Information). The salt bridges at the ubiquitin/ RASIP1 interface generated interfacial electrostatic forces, contributing ≈245.0 kcal mol^−1^ interaction energies, which are comparable to the RAP1A‐G12V/ RASIP1 interaction energies (≈315.2 kcal mol^−1^). This indicates that RAP1A ubiquitination might enhance the RAP1A‐G12V/ RASIP1 interactions due to the favorable ubiquitin/ RASIP1 interface.

In contrast, the simulations of the ubiquitin‐RAP1A‐G12V/ TLN1 complex formation showed that ubiquitin failed to form a strong interface with TLN1 (Figure S5e,f, Supporting Information). In model 1, the ubiquitin formed only one salt bridge with TLN1 and contributed ≈−160.7 kcal mol^−1^ interaction energy, which was less than its contribution to the ubiquitin‐RAP1A‐G12V/ RASIP1 complex. The differential effect of RAP1 ubiquitination on its binding to RASIP1 or TLN1 proteins likely comes from the different surfaces of the downstream effectors to accommodate ubiquitin. Both RASIP1 and TLN1 mostly used the α‐helical motif (residues 172–182 in RASIP1 and residues 25–35 in TLN1) to interact with ubiquitin. Whereas RASIP1 has three negatively charged residues, E174, E178, and E181, to establish an intense acidic surface for ubiquitin interactions, TLN1 has R30 in the middle that would interfere with binding to ubiquitin (Figure 3c). Furthermore, in model 2, TLN1 showed impaired interaction with ubiquitinated RAP1. Altogether, these results suggest that ubiquitination suppresses the interaction between RAP1A‐G12V and TLN1 (Figure S5g,h).

In line with in silico modeling, the PLA and the immunoprecipitation experiments demonstrated that LSS, which induced RAP1 ubiquitination, led to an increase in the binding of RAP1 to RASIP1 but decreased RAP1 binding to TLN1 (Figure 3d,e; Figure S6a, Supporting Information). The ubiquitination‐deficient RAP1‐K31R mutant showed decreased the binding of RAP1 to RASIP1 in YODA1‐treated HUVECs when compared to wt‐RAP1 (Figure 3f). Furthermore, RAP1 showed higher affinity to TLN1 and lower affinity to RASIP1 in the *Wwp2* knockout sorted endothelial cells (Figure 3g). Altogether, these results indicate that WWP2‐mediated ubiquitination of RAP1 at K31 switches the balance from the RAP1/ TLN1 toward the RAP1/ AFDN or RAP1/ RASIP1 complex formation.

### 2.5 WWP2‐Mediated RAP1 Ubiquitination Maintains Endothelial Homeostasis

As a next step, we assessed the role of WWP2‐mediated RAP1 ubiquitination at K31 in maintaining vascular homeostasis. Both RAP1/ AFDN and RAP1B/ RASIP1, a part of a bigger HEG1/ KRIT1/ RAP1/ RASIP1 complex, are essential for the stabilization of endothelial junctions.^[^ ^23^, ^42^, ^43^ ^]^ Thus, we first assessed the contribution of WWP2‐mediated RAP1 ubiquitination at K31 to LSS‐mediated cytoskeleton re‐organization. Either *WWP2* suppression or the RAP1‐K31R mutation impaired the junctional integrity of HUVECs under long‐term LSS (Figure 3h), suggesting that RAP1B ubiquitination is crucial for the stabilization of adherens junctions. Concordantly, the permeability assay revealed an increase in permeability of the monolayer of HUVECs depleted for *WWP2* or expressing the ubiquitination‐deficient RAP1B‐K31R mutant when compared to the respective controls (Figure 3i).

**Figure 4:**
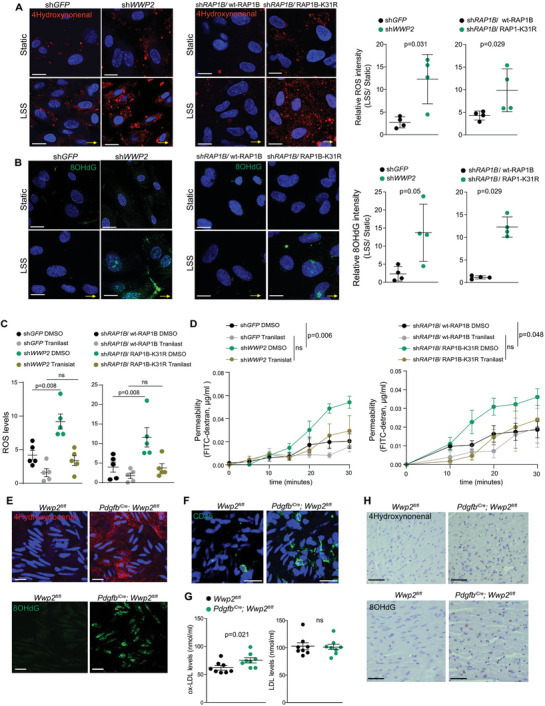
WWP2‐mediated RAP1 ubiquitination on K31 is essential for maintaining endothelial cell homeostasis. a,b) Levels of lipids peroxidation and oxidative DNA damage in static or shear stress‐activated HUVECs expressing the indicated constructs. HUVECs were exposed to LSS, 6 dyn cm^−2^, 24 h, and stained for 4‐Hydroxynonenal (a) or 8‐hydroxy‐2′‐deoxyguanosine (8‐OHdG) (b). Scale bar, 10 µm. Yellow arrows show the direction of flow. Intensity levels are shown as mean ± SEM. *N* = 4. *p*‐values, the Wilcoxon Mann–Whitney test. c) Relative ROS levels after 1 h YODA1 (3 µm) activation measured by Cell‐ROX in HUVEC cells expressing the indicated constructs and treated with vehicle or Tranilast (1 µm). Data is shown as mean ± SEM. *N* = 5 each. *p*‐values, the Wilcoxon Mann–Whitney test. d) FITC‐Dextran permeability of the monolayer of HUVECs expressing the indicated constructs and treated with vehicle or Tranilast (1 µm). Data is shown as mean ± SEM. *N* = 5. *p*‐values, the two‐way ANOVA. e) Staining for 4‐Hydroxynonenal and 8‐OHdG of descending aorta cryosection of *Wwp2* ^*fl/fl*^ and *Pdgfb* ^*iCre*^; *Wwp2* ^*fl/fl*^ injected with tamoxifen at weaning. Scale bar, 20 µm. f) CD45 immunostaining of en face aorta of *Wwp2* ^*fl/fl*^ and *Pdgfb* ^*iCre*^; *Wwp2* ^*fl/fl*^ mice at age 15–16 weeks. Scale bar, 20 µm. g) Levels of LDL and OX‐LDL in the blood of Wwp2^fl/fl^ and Pdgfb^iCre^; Wwp2^fl/fl^ mice injected with tamoxifen at weaning determined by ELISA. Data are shown as mean ± SEM. *N* = 7–8 per group. *p*‐value, the Wilcoxon Mann–Whitney test. h) Staining for 4‐Hydroxynonenal and 8‐OHdG of heart ventricle of *Wwp2* ^*fl/fl*^ and *Pdgfb* ^*iCre*^; *Wwp2* ^*fl/fl*^ mice at age 15–16 weeks. Scale bar, 50 µm.

Multiple reports also demonstrated that either activated RAP1 or KRIT1 contributes to endothelial barrier integrity by inhibiting ROS production through the regulation both NADPH oxidase and NF‐kβ transcriptional activity.^[^ ^44^, ^45^, ^46^ ^]^ Thus, we examined whether RAP1 ubiquitination contributes to the regulation of the endothelial barrier function by suppressing ROS levels. We found that LSS‐stressed HUVECs expressing either sh*WWP2* or the RAP1‐K31R mutant showed higher levels of ROS, peroxidized lipids, and oxidative DNA damage (**Figure** **4**a,b,c), indicating that the dysregulation of RAP1 ubiquitination results in increased ROS production. Given that a previous study demonstrated that treatment with Tranilast, an antioxidant and immunosuppressive drug,^[^ ^47^ ^]^ rescued barrier function in *KRIT1*‐deficient cells dysregulated by high ROS production,^[^ ^48^ ^]^ we examined the effect of Tranilast in our system. Similar to the effect observed in *KRIT1*‐deficient cells, Tranilast treatment normalized ROS levels and reduced permeability in HUVECs with *WWP2* knockdown or expression of the RAP1‐K31R mutant (Figure 4c,d). These results indicate that WWP2‐mediated RAP1 ubiquitination is essential to maintain endothelial junctional integrity by buffering ROS production.

We also detected the accumulation of peroxidized lipids, increased oxidative DNA damage, and inflammation in the endothelial layer of the descending aorta of *Pdgfb‐Cre* ^*ERT2*^, *Wwp2* ^*fl* */* *fl*^ mice (Figure 4e,f), a region where we observed a spatial dysregulation of RAP1 ubiquitination (Figure 2j,k). Concordantly, we observed increased oxidized low‐density lipoprotein (OX‐LDL) levels in the blood of *Pdgfb‐Cre* ^*ERT2*^, *Wwp2* ^*fl/fl*^ mice, while not detecting any major changes in total low‐density lipoprotein (LDL) level (Figure 4g). Finally, we observed higher levels of peroxidized lipids and oxidative DNA damage in the heart tissue of *Wwp2* knockout mice (Figure 4h). Altogether, these results indicate that the WWP2‐mediated RAP1 ubiquitination is required to suppress ROS production and LSS‐induced inflammation.

### 2.6 Discussion

Given that shear stress coordinates multiple interconnected intracellular processes, unbiased systems biology‐based and spatial approaches are essential to understanding vascular physiology. Here, we employed unbiased proteomic approaches to decipher the role of the ubiquitin system in endothelial shear stress response, as the ubiquitin enzymes have the major advantage of being “druggable”, making them promising targets for the modulation of endothelial homeostasis, especially in the context of cardiovascular diseases.

Ubiquitome analysis revealed that shear stress promotes the non‐degradative ubiquitination of intracellular GTP‐binding proteins, including both heteromeric G‐proteins and small GTPases. Previous studies suggested that non‐degradative ubiquitination could control the function of small GTPases identified in our OMICs analysis, by altering their interaction network.^[^ ^20^ ^]^ In line with these reports, we observed that the non‐degradative ubiquitination of RAP1 fine‐tunes its interaction affinity with effectors. These in vitro findings were confirmed by assessing RAP1 ubiquitination spatially in aortic vascular beds exposed to differential shear stresses.

Whilst GTP binding is crucial to activate RAP1, the ubiquitination at K31, which is located within the RAP1 effector‐binding domain, tunes its affinity toward specific effector proteins. Specifically, under long‐term LSS, RAP1 ubiquitination at K31 promotes the assembly of RAP1 complexes with AFDN and RASIP controlling cell‐cell junction formation and endothelial barrier integrity. On the other hand, ubiquitinated RAP1 has a lower affinity to TLN1, a component of the multiprotein adhesion complexes.^[^ ^49^ ^]^ While the dysregulation of RAP1 ubiquitination impaired the junctional integrity, it only moderately affected the elongation of vinculin filaments (Figure S6B, Supporting Information). Live imaging of focal adhesion filaments using GFP‐TLN1 revealed that long‐term LSS led to the formation of mature stable focal adhesions (Figure S6C, Supporting Information). Based on these observations, we could speculate that RAP1 activity is required for both the formation and stabilization of adherent junctions by interacting with RASIP1 and AFDN. On the other hand, whereas RAP1/ TLN1 activity is essential at the initial stage of shear stress response during the formation of the focal adhesions, it is not required for the stabilization of mature focal adhesions under long‐term LSS conditions. This suggests a novel molecular loop by which shear stress regulates adhesion and junctional plasticity in endothelial cells through RAP1 ubiquitination.

WWP2, which has a membrane‐binding Ca^2+^/phospholipid‐binding C2 domain,^[^ ^50^ ^]^ is known to regulate various physiological and pathological activities by ubiquitinating multiple membrane targets, such as Dishevelled‐3,^[^ ^51^ ^]^ PTEN,^[^ ^52^ ^]^ α‐arrestin/ ARRDC3,^[^ ^53^, ^54^ ^]^ and ALIX/ P2Y1.^[^ ^55^ ^]^ Here, we found that WWP2 specifically ubiquitinates RAP1 in endothelial cells, exposed to physiological laminar shear stress, to maintain endothelial homeostasis. Even though WWP2 has been shown to regulate the ubiquitination of multiple substrates, among proteins showing differential ubiquitination in response to YODA1 activation, only RAP1 is a predicted WWP2 substrate, according to the Ubibrowser. Depleting *WWP2* or expressing a ubiquitination deficient RAP1 mutant led to similar pathogenic responses to shear stress. Moreover, loss of *Wwp2* in endothelial cells affected RAP1 ubiquitination as assessed by spatial analysis, leading to inflammation, and accumulation of oxidized lipids in specific vascular beds exposed to laminar shear stress, such as the descending aorta. This is in line with a recent study showing a predisposition to aortic inflammation in the *Rap1* endothelial knockout mice.^[^ ^56^ ^]^ This data strongly demonstrates that RAP1 is one of the major substrates of WWP2 in endothelial cells in response to shear stress.

## 3 Conclusion

Our study provides a global view and a mechanistic explanation for the contribution of the ubiquitin system during the shear stress response, highlighting the complexity of mechano‐sensing signaling dynamically and spatially. While it has been known for decades that GTPases are essential to promote mechanobiological responses and that their functions are vital for normal cardiac function, targeting them directly remains extremely challenging. This work uncovers the ubiquitin landscape of endothelial mechanoactivation, revealing a list of proteins modified by ubiquitination upon YODA1 treatment. Such work is essential to provide a better understanding of mechanoresponse in endothelial cells, as ubiquitination can affect protein stability, trafficking, function, or activity. On the other hand, identification of regulators of such ubiquitination processes, such as WWP2, is essential for the development of novel therapies in pathologies where blood flow is altered, as drugs blocking disease‐specific posttranslational modifications such as protein ubiquitination could have low toxicity compared to conventional drugs, and be beneficial for patients with cardiovascular diseases, where therapeutic options are often limited.^[^ ^57^ ^]^ Further studies are necessary to investigate the therapeutic potential of these novel regulators of endothelial response to flow.^[^ ^58^ ^]^

## 4 Study Limitations

The use of YODA1 as a mimic of shear stress has obvious limitations since the response of endothelial cells to shear stress involves many mechanosensors, of which PIEZO1 is only one. This was necessary because of the large amount of protein needed for the unbiased proteomics screen for ubiquitin modifications. We observed similar profiles for RAP1 and calcium signaling activation as well as for ROS levels in HUVECs treated with YODA1 or exposed to LSS, indicating a phenotypic overlap between both treatments. While we confirmed the contribution of RAP1 ubiquitination in shear stress response, we cannot extend this to all proteins identified in the screen, nor can we exclude that we missed proteins that would be ubiquitinated by physiological shear stress.

We also limited our study to steady laminar shear stress, since YODA1 cannot mimic more complicated pathophysiological flows that would be of interest in terms of druggable targets.

Furthermore, laminar shear stress does not capture the full complexity of the mechanical forces that the endothelium encounters in vivo. Indeed, the laminarity of blood flow is a simplification as blood flow can be oscillatory, pulsatile, or turbulent, depending on the type of vessel, the presence of bifurcations, aneurysms, or atherosclerosis. These different types of flow have different effects on the endothelium. Therefore, using laminar shear stress in a 2D culture system, with a cell culture media fluid that does not recapitulate blood composition and viscosity, as a readout for the complexity of endothelial mechanosensing has limitations, as it does not faithfully reproduce the physiological and pathological conditions encountered by the endothelium in the vascular system. However, comparing laminar flow to static conditions is an acceptable simplification of very complex physical‐biological processes that cannot be fully understood yet. This is why we validated most of our findings on the endothelial layer of the aorta and sorted endothelial cells from mice presenting endothelial deletion of *Wwp2*. The fact that similar phenotypes were observed with YODA1 treatment, laminar shear stress in vitro, and in the endothelial cells in vivo, confirms the robustness and relevance of our findings.

## Conflict of Interest

The authors declare no conflict of interest.

## Author Contributions

E.J. and A.S. contributed equally to this work. R.N.S. genetically engineered the mouse model and performed protein assays. P.C. analyzed the hearts of the mice. F.R. performed the assays using HEK293T cells. T.I. performed the histology. M.Z. and H.J. performed the in‐silico modeling under the supervision of R.N. P.Z. performed the bioinformatic analysis. B.L. performed the genotyping of mice and the cloning of plasmids. D.V.H. performed the mass spectrometry analysis under the supervision of F.I. E.A.J. performed the shear stress experiments. R.N.S., E.A.J., and A.A.S. designed the study. R.N.S., E.A.J., and A.A.S. analyzed the data and wrote the manuscript. All authors discussed and commented on the manuscript.

## Supporting information

Supporting Information

## Data Availability

The data that support the findings of this study are available from the corresponding author upon reasonable request.
