# Supplementary material for: Spatial Mechano‐Signaling Regulation of GTPases through Non‐Degradative Ubiquitination
Source: Adv Sci (Weinh). 2023 Nov 9;10(36):2303367. doi: 10.1002/advs.202303367 (PMC10754123; doi:10.1002/advs.202303367)
Supplement: Supplementary file 1 — Supporting Information [file ADVS-10-2303367-s001.pdf]

ADVANCED SCIENCE
Open Access

Supporting Information

for *Adv. Sci.*, DOI 10.1002/advs.202303367

Spatial Mechano-Signaling Regulation of GTPases through Non-Degradative Ubiquitination

*Raj N. Sewduth, Paolo Carai, Tonci Ivanisevic, Mingzhen Zhang, Hyunbum Jang, Benoit Lechat, Delphi Van Haver, Francis Impens, Ruth Nussinov, Elizabeth Jones and Anna Sablina**

Supplemental Materials and Methods

Genetically modified mice

All procedures involving animals were performed in accordance with the guidelines of the IACUC of KU Leuven and approved in project application P143/2016. B6.129S-*Wwp2*$^{tm1(flox)Smoc}$ mice were purchased from Shanghai model organism. To determine the number of required animals, a power analysis was conducted using the findings of a previous study[1]. Interbred, homozygous *Wwp2*$^{fl/fl}$ mice exhibit normal development and are viable/ fertile. For endothelial cell-specific deletion, *Pdgfb*^{iCreERT2} transgenics (from M. Fruttiger laboratory, University College London) were bred in a background of *Wwp2*$^{fl/fl}$ mice. *Pdgfb*^{iCreERT2}, *Wwp2*$^{fl/+}$ males were then mated with *Wwp2*$^{fl/+}$ females. Tamoxifen injection (Sigma Aldrich 10 mg/ml; 100 µl for mouse 25 g) was performed on pregnant females at E8.5, E10.5, and E12.5 or 5 times on pups at weaning (age 3 weeks old, 5 consecutive days, alternating injection side). The cardiovascular phenotypes in adult mice were assessed 14 weeks after tamoxifen injection.

Cell culture

Endothelial cells were sorted from different tissues of pups after homogenization and digestion with a Multi Tissue Dissociation Kit 2 (Miltenyi Biotech, 130-110-203). The digested mix of cells was first incubated with CD45 and the flow-through with CD31 magnetic beads (Miltenyi Biotech). The quality of each fraction was evaluated by RT-qPCR, immunostaining, or immunoblotting for CD31 and VE-Cadherin.

Four independent pools of HUVECs were purchased from Promocell and cultured according to the manufacturer's guidelines. HUVECs were treated with 3 µM YODA1 (SML1558, Sigma-Aldrich) for 48h, or 1µM Tranilast ((2-([(2E)-3-(3,4-dimethoxyphenyl)prop-2-

enoyl]amino)benzoic acid, Rizaben) (T0318, Sigma-Aldrich). The CellROX® Green Reagent (C10444, ThermoFisher) was used as recommended by the supplier.

HUVECs were seeded on collagen coated Ibidi chambers (micro-slide VI^0.4). Cells were returned to the tissue culture incubator for 24 hours with a media change after 10 hours, so that endothelial cells could adhere. Cells were then sheared using a peristaltic pump (Masterflex LS 7550-30) in starvation media (Promocell, ECGM MV2 with only 0.5% FBS and no growth factors). For static wells, media was changed to starvation media and refreshed once. Lentiviral infections were performed as described by the RNAi Consortium (TRC) (http://www.broadinstitute.org/rnai/public/resources/protocols). The generated viruses were titrated using the Lenti-X™ p24 Rapid Titer Kit (Takara Bio). A Multiplicity of Infection (MOI) of 10 to 30 was used to infect the HUVECs.

A parallel plate flow chamber was designed in-house for the collection of cells for immunoblotting and immunoprecipitation. The parallel plate flow chamber was connected to a closed-loop perfusion system consisting of a vented media reservoir, a flow dampener, a Masterflex 7550-30 peristaltic pump with Easy-load II pump head. The chamber has a height of 300 µm and a width of 28 mm. Cells were seeded on collagen coated Superfrost Excell microscope slides and never seeded cells on the slide labelling area (white) nor within 5mm of any edge. Slides were oriented within the chamber so that the labelling area was on the side of the inlet of the flow. HUVECs were exposed to laminar flow at a calculated wall shear stress of 6 dynes/cm2. Shear stress is calculated based on Tau = 6*µ*Q/bh^2; where µ is the viscosity, Q is the volumetric flow rate, b is the channel width and h is the channel height. Static slides were cultured in parallel and given fresh media. Calculations for this system were done using the information provided by: https://ibidi.com/img/cms/support/AN/AN11_Shear_stress.pdf

To investigate the permeability of the HUVEC monolayer, an assay was established based on previous reports[2]. After plating HUVECs on 0.4 µm pore size inserts (Transwell, BD

Biosciences), the inserts were placed into a 12-well plate. Following a 3-day culture, the HUVECs formed a monolayer, and the medium in the inserts was aspirated to be replaced with cell-culture media containing 25 µg/mL of 40 kDa FITC-dextran dye (Sigma Merck) in each reservoir. A fraction of media from the bottom chamber was collected every 5 minutes to measure the amount of FITC-dextran using a VICTOR NIVO reader. This amount was normalized to establish a permeability curve from 0 to 30 minutes after FITC-dextran addition.

Plasmids

Two different shRNA targeting *WWP2* were purchased from Sigma (MISSION® shRNA, TRCN0000001514 and TRCN0000001515). The pMT107–6×His–ubiquitin plasmid was a generous gift from Dr. Bohmann (University of Rochester, USA). Complementary DNA of WWP2 (#325) originated from the Orfeome collection; RAP1A (SC127673) and RAP1B (SC114605) cDNAs were from Origene. cDNAs were cloned into pLA-CMV vector with N-terminal HA or Flag tags. WWP2-C838A, RAP1B-K5R, RAP1B-K31R, RAP1B-G12V, RAP1B-G12V/K31R point mutations were introduced by using site-directed mutagenesis. shRNA targeting *RAP1* and HA-tagged *RAP1B* wt or K31R coding sequence were cloned in a pLKO.1 plasmid to generate the shRNA *RAP1B*- HA- *RAP1B* WT and shRNA *RAP1B*- HA- *RAP1B* K31R respectively. The *RAP1B* wt or K31R coding sequence was optimized so that the shRNA does not recognize it.

Sequence of sh *RAP1B*: *GCCCAGCAATGCATGCTCGAG*

Codon-optimized *RAP1B* WT sequence:

ATGCGAGAATACAAGCTCGTAGTATTGGGGAGTGGAGGAGTGGGTAAATCAGCACTCA CAGTGCAGTTCGTTCAAGGAATTTTCGTGGAAAAGTATGACCCGACTATCGAGGACTCA TACCGAAAACAAGTTGAAGTCGATGCCCAGCAATGCATGCTCGAGATACTGGACACCG CCGGGACGGAGCAATTTACAGCCATGAGGGACCTTTACATGAAGAATGGGCAAGGATT

TGCATTGGTCTACAGTATAACTGCACAGTCTACCTTTAACGACTTGCAAGACCTCAGGG AGCAAATACTGCGCGTCAAAGATACCGACGACGTGCCTATGATACTCGTCGGCAACAA GTGTGACCTTGAAGATGAGCGCGTCGTAGGGAAGGAACAGGGTCAGAATCTTGCTCGA CAATGGAATAACTGCGCCTTCCTTGAGAGTAGCGCCAAATCAAAAATAAATGTCAACGA GATTTTCTACGACTTGGTACGCCAGATCAATCGCAAGACCCCTGTACCTGGCAAAGCAC GCAAGAAGTCATCTTGTCAGCTTCTTTAA

Antibodies

The following antibodies/ dyes were used:

Mouse monoclonal	Anti RAP1 (Santa Cruz Biotechnology, sc-398755), anti Vinculin (Sigma Aldrich, clone hVIN-1, V 9131),
Rabbit monoclonal	Anti VE-cadherin (Cell Signaling, 2500P), anti TLN1 (Cell Signaling, C45F1), anti-RAP1B (Cell Signaling, #2326),
Rabbit polyclonal	Anti-β–actin (Sigma Aldrich, A2066), anti-Ubiquitin (Abcam, ab7780), anti WWP2 (Bethyl, A302-935A), AFDN (Proteintech, 55102-1-AP), RASIP1 (Proteintech, 26064-1-AP), RADIL (Proteintech, 20284-1-AP),
Rat monoclonal	anti CD45 (Santa Cruz Biotechnology, sc-53665),
Alexa 647-coupled dyes/ antibodies	Vinculin (Santa Cruz Biotechnology, sc-73614).

The specificity of the WWP2 antibody was validated by performing immunostaining and immunoblotting using *Wwp2*-depleted cells. The following secondary antibodies were used: Donkey or Goat Alexa488-or Alexa568 or Alexa594- or Alexa 647-conjugated secondary antibodies (Thermo Fisher, Molecular Probes) (A10037, A10042, A11005, A11006, A11012, A11034, A11057, A21201, A21245, A21247, A21447) or HRP-labelled antibodies (DAKO) (P0447, P0448).

Ubiquitome analysis

HUVEC cell pellets were homogenized in 10 ml urea lysis buffer containing 9 M urea and 20 mM HEPES pH 8.0. The samples were sonicated with 3 pulses of 15 s at an amplitude of 20% using a 3 mm probe, with incubation on ice for 1 minute between pulses. After centrifugation for 15 minutes at 20,000 x g at room temperature to remove insoluble components, proteins were reduced by addition of 5 mM DTT and incubation for 30 minutes at 55˚C and then alkylated by addition of 10 mM iodoacetamide and incubation for 15 minutes at room temperature in the dark. The protein concentration was measured using a Bradford assay (Bio-rad) and from each sample, 30 mg protein was used to continue the protocol. Samples were further diluted with 20 mM HEPES pH 8.0 to a final urea concentration of 4 M and proteins were digested with 75 µg LysC (Wako) (1/400, w/w) for 2 hours at 37°C. Samples were again diluted to 2 M urea and digested with 75 µg trypsin (Promega) (1/400, w/w) overnight at 37˚C. The resulting peptide mixture was acidified by addition of 1% trifluoroacetic acid (TFA) and after 15 minutes incubation on ice, samples were centrifuged for 15 minutes at 1,780 x g at room temperature to remove insoluble components. Immunocapture of GlyGly-modified peptides was then performed using the PTMScan® Ubiquitin Remnant Motif (K-ε-GG) Kit (CST) according to the manufacturer's instructions. Briefly, peptides were purified on Sep-Pak C18 cartridges (Waters), lyophilized for 2 days, and re-dissolved in 1.45 ml 1x immunoprecipitation buffer supplied with the kit. Note that at this point, 50 µl aliquots of digested protein material were taken and desalted on reversed-phase C18 OMIX tips (Agilent), according to the manufacturer's protocol for shotgun proteomics analysis. Peptides were incubated with the antibody-bead slurry for 2 h on a rotator at 4°C and after several wash steps, GlyGly-modified peptides were eluted in 100 µl 0.15% TFA and also desalted on reversed-phase C18 OMIX tips, all according to the manufacturer's protocol. Purified shotgun and GlyGly-modified peptides were dried under vacuum in HPLC inserts, and stored at −20°C until LC-MS/MS analysis.

Purified peptides for shotgun analysis were re-dissolved in 20 µl solvent A (0.1% TFA in water/ACN (98:2, v/v) and peptide concentration was determined by measuring on a Lunatic spectrophotometer (Unchained Labs). 2 µg of each sample was injected for LC-MS/MS analysis on an Ultimate 3000 RSLCnano system in-line connected to a Q Exactive HF mass spectrometer equipped with a Nanospray Flex Ion source (Thermo). Trapping was performed at 10 µl/min for 4 min in solvent A on a 20 mm trapping column (made in-house, 100 µm internal diameter (I.D.), 5 µm beads, C18 Reprosil-HD, Dr. Maisch, Germany) and the sample was loaded on a 200 cm long micropillar array column (PharmaFluidics) with C18-end-capped functionality mounted in the Ultimate 3000's column oven at 50°C. For proper ionization, a fused silica PicoTip emitter (10 µm I.D., New Objective) was connected to the µPAC™ outlet union and a grounded connection was provided to this union. Peptides were eluted by a non-linear increase from 1 to 55% MS solvent B (0.1% FA in water/ACN (2:8, v/v)) over 145 minutes, first at a flow rate of 750 nl/min, then at 300 nl/min, followed by a 15-minutes wash reaching 99% MS solvent B and re-equilibration with MS solvent A (0.1% FA in water). The mass spectrometer was operated in data-dependent mode, automatically switching between MS and MS/MS acquisition for the 16 most abundant ion peaks per MS spectrum. Full-scan MS spectra (375-1,500 m/z) were acquired at a resolution of 60,000 in the Orbitrap analyzer after accumulation to a target value of 3E6. The 16 most intense ions above a threshold value of 1.3E4 (minimum AGC of 1E3) were isolated for fragmentation at a normalized collision energy of 28%. The C-trap was filled at a target value of 100,000 for a maximum 80 ms and the MS/MS spectra (200-2,000 m/z) were acquired at a resolution of 15,000 in the Orbitrap analyzer with a fixed first mass of 145 m/z. Only peptides with charge states ranging from +2 to +6 were included for fragmentation and the dynamic exclusion was set to 12 s.

Purified GlyGly-modified peptides were re-dissolved in 20 µl solvent A of which 15 µl was injected for LC-MS/MS analysis on the same setup as for the shotgun samples. Peptides were

eluted by a nonlinear increase from 1 to 55% MS solvent B over 116 minutes, first at a flow rate of 750 nl/min, then at 300 nl/min, followed by a 14-minutes wash reaching 99% MS solvent B and re-equilibration with MS solvent A. The mass spectrometer was operated in data-dependent mode, automatically switching between MS and MS/MS acquisition for the 8 most abundant ion peaks per MS spectrum. Full-scan MS spectra (375-1500 m/z) were acquired at a resolution of 60,000 in the orbitrap analyzer after accumulation to a target value of 3E6. The 8 most intense ions above a threshold value of 8.3E3 were isolated (window of 1.5 Th) for fragmentation at a normalized collision energy of 28% after filling the trap at a target value of 1E5 for a maximum of 120 ms. MS/MS spectra (200-2000 m/z) were acquired at a resolution of 15,000 in the orbitrap analyzer. Only peptides with charge states ranging from +2 to +6 were included for fragmentation and the dynamic exclusion was set to 12 s. QCloud was used to control instrument longitudinal performance during the project.

Data analysis was performed with MaxQuant (version 1.6.3.4) using the Andromeda search engine with default search settings including a false discovery rate set at 1% on both the peptide and protein level. Two different searches were performed to analyze the spectra from the GlyGly-enriched samples and the shotgun samples. In both searches, spectra were interrogated against the human proteins in the Swiss-Prot Reference Proteome database (database release version of January 2019 containing 21,074 human protein sequences, (http://www.uniprot.org)). The mass tolerance for precursor and fragment ions was set to 4.5 and 20 ppm, respectively, during the main search. Enzyme specificity was set as C-terminal to arginine and lysine, also allowing cleavage at proline bonds with a maximum of three missed cleavages. Variable modifications were set to oxidation of methionine residues, acetylation of protein N-termini and GlyGly modification of lysine residues, while carbamidomethylation of cysteine residues was set as fixed modification. Matching between runs was enabled with a matching time window of 0.7 minutes and an alignment time window of 20 minutes. Only

proteins with at least one unique or razor peptide were retained leading to the identification of 3,960 proteins and 9,535 GlyGly modified peptides. Proteins were quantified by the MaxLFQ algorithm integrated in the MaxQuant software. A minimum ratio count of two unique or razor peptides was required for quantification.

Further data analysis of the shotgun results was performed with the Perseus software (version 1.6.2.1) after loading the protein groups file from MaxQuant. Reverse database hits were removed, LFQ intensities were log2 transformed and replicate samples were grouped. Proteins with less than three valid values in at least one group were removed and missing values were imputed from a normal distribution around the detection limit leading to a list of 2,780 quantified proteins that were used for further data analysis and to compare control and YODA-1-treated samples (t-test with FDR=0.05 and s0=1). For the analysis of the GlyGly modified peptide data, the GlyGly(K)Sites file was loaded in the Perseus software (version 1.6.2.1). Reverse hits were removed, the site table was expanded, the intensity values were log2 transformed and the median was subtracted. Replicate samples were grouped, GlyGly(K)sites with less than three valid values in at least one group were removed and missing values were imputed from a normal distribution around the detection limit leading to a list of 927 quantified GlyGly peptides that were used for further data analysis. Then, a t-test was performed (FDR=0.05 and s0=1) to compare control and YODA-1-treated samples. The mass spectrometry proteomics data have been deposited to the ProteomeXchange Consortium via the PRIDE partner repository with the dataset identifier PXD024309 (login username: reviewer_pxd024309@ebi.ac.uk and password: KSwemKZU).

Cellular and tissue staining

For immunostaining, $2x10^4$ cells were plated on an 8-well chamber glass slide (Ibidi) and fixed with 4% paraformaldehyde in PBS or ice-cold methanol. Cells were permeabilized in PBS-0.15% Triton-X100 and blocked with 1% BSA and 10% horse serum. Primary antibodies and

donkey Alexa-conjugated secondary antibodies were applied diluted in a blocking buffer before mounting with Vectashield (Vector Laboratories). A negative control (a secondary antibody without primary) was used to distinguish genuine target staining from the background.

Tissue was fixed in 4% paraformaldehyde overnight and processed for paraffin embedding. Hematoxylin and eosin-stained sections (4 µm) were used to assess overall morphology, and Masson trichome staining for overall fibrosis. For immunohistochemistry, paraffin slides were first rehydrated to proceed further with antigen retrieval in citrate solution (DAKO). If necessary, 0.3% H_2O_2 was added to methanol to block endogenous peroxidases. For immunofluorescence, Vectashield mounting medium (Vector) was used. Otherwise, 3,39-diaminobenzidine (DAB) (Vector) was used as a detection method followed by Harris' counterstaining, dehydration, and mounting with DPX mounting solution (Sigma-Aldrich). For *en face* aorta staining, thoracic aortas were collected after 4% PFA perfusion and fixation of the heart/ aorta/ ribcage in 2% PFA for 60 min. The aortas were then cut open (aortic arch or descending aorta) and stained as described in previous papers [3].

Microscopic analysis

Microscopic analysis was done with a Leica DCF-6000 confocal microscope; images were acquired with LAS X software. Some images were acquired using a ZEISS Axiobserver and acquired using ZEN Blue (ZEISS). Image analysis was performed with Imaris (Bitplane) or ImageJ. Imaged fields for quantification were randomly selected on the tissue section/ cell monolayer. Representative images were chosen after the quantification, as the most accurate depiction of the observed phenotypes. The Cell-light TLN1 BACMAM (Thermofisher, C10611) was used according to the manufacturer's instructions for live imaging of Talin structures (every 10 minutes for 60 minutes). Quantification of TLN1 structures was performed by surfacing the structures at different time points of the acquisition and the stability was calculated automatically in the Surpass mode from Bitplane Imaris. Quantification of Proximity

ligation assay signal was performed by surfacing each dot and each nucleus per field, and then making a ratio of the number of vesicles per nuclei using the "Surpass mode" of Bitplane Imaris, giving an average number of PLA positive dots per cell. A minimum of 5 fields was quantified per condition with a minimum of 20 cells per field. Quantification of Signal Intensity was performed using the "Surpass mode" of Bitplane Imaris, giving a mean intensity value per field. A minimum of 5 fields and 20 cells per field was quantified per condition. Quantification of Junctional Gaps and filament elongation was performed using the "Surpass mode" of Bitplane Imaris, after surfacing of the structures. A minimum of 5 fields and 20 cells per field was quantified per condition.

Immunoprecipitation, immunoblotting, and electrochemiluminescent immunoassay

Cells were washed twice in cold PBS and scraped on ice in lysis buffer (50 mM Tris-HCl pH 7.5, 150 mM NaCl, 1% NP-40) containing protease inhibitor, phosphatase inhibitor cocktails (Roche) as well as a De-Ubiquitinase inhibitor Cocktail (Signal-Seeker™ Ubiquitination Detection Kit, Cytoskeleton Inc). Samples were subsequently cleared by centrifugation for 10 minutes at 16,000 g at 4°C. Proteins were immunoprecipitated using anti-HA agarose beads (Sigma-Aldrich, A2095), anti-ubiquitin FK2 agarose beads (MBL, D058-8), anti-RAP1 agarose beads (Santa Cruz, Clone E6, sc-398755 AC) for 4 hours at 4°C, washed five times with cold co-immunoprecipitation buffer and finally eluted according to the manufacturer's protocol. For immunoblotting, equivalent amounts of cell lysates were separated on 4-12% gradient gels (Invitrogen), transferred to nitrocellulose membranes, and incubated with the indicated antibodies. The signal was visualized by chemiluminescence (Amersham Pharmacia Biotech) using an automated digital developer.

Purification of ubiquitinated proteins.

HEK293T cells were co-transfected with 6xHis–ubiquitin and Flag–RAP1B. Ubiquitinated proteins were purified as described previously [4]. Briefly, cells were lysed in a co-

immunoprecipitation buffer containing an EDTA-free protease inhibitor cocktail (Roche). Cell lysates were mixed with His-buffer A (PBS, at pH 8.0, 6 M guanidinium-HCl, 0.1% NP-40, and 1 mM β-ME) at proportion 1:10 and added to TALON beads (Clontech). After binding, the resin was washed with His-buffer B (PBS, at pH 8.0, 0.1% NP-40, 5% glycerol, and 20 mM imidazole), and proteins were eluted in the LDS sample buffer.

For TAP purification, HEK293T cells were co-transfected with Flag–RAP1B and 6xHis–ubiquitin. Flag–RAP1B was immunoprecipitated using anti-Flag (M2) agarose (Sigma-Aldrich), washed twice with lysis buffer, once with buffer containing 50 mM Tris, at pH 7.5, 100 mM LiCl, and eluted with 3xFlag peptide. Ubiquitinated Flag–RAP1B was then purified using TALON beads (Clontech) as described above.

RNA expression analysis

For mRNA analysis, the tissues were homogenized using a Precellys tissue homogenizer, and RNA was then extracted using the Nucleospin RNA extraction kit (Macherey Nagel). RNA from cultured cells was extracted with the Nucleospin RNA extraction kit. Reverse transcription to cDNA was performed with the SuperScript III First-Strand cDNA Synthesis Kit (Life Technologies). Primers for qPCR were designed using Primer bank (Harvard). cDNA, primer mix and Sybr Green Mix were prepared according to the manufacturer's instructions (Roche). Samples were analyzed by a Light Cycler 96 (Roche).

Human Primers	Sequence
hWWP2 FW	CAAAGCCCAAGGTGCATAATCG
hWWP2 RV	CCAATGCGCTTCCCAGTCT
hRAP1B FW	AGC AAG ACA ATG GAA CAA CTG T
hRAP1B RV	TGC CGC ACT AGG TCA TAA AAG
hRCAN1 FW	GCG TGG TGG TCC ATG TAT GT
hRCAN1 RV	TGA GGT GGA TCG GCG TGT A
hHMBS FW	GGGTACCCACGCGAATCAC
hHMBS RV	GGCAATGCGGCTGCAA

hACTN FW	CTGGGACGACATGGAGAAAA
hACTN RV	AAGGAAGGCTGGAAGAGTGC
hB2M FW	TGCTGTCTCCATGTTTGATGTATCT
hB2M RV	AAATGGTTGAGTTGGACCCGA

**Mouse Primers**	**Sequence**
mDnm1 FW	AATATGCCGAGTTCCTGCACT
mDnm1 RV	GTCTCAGCC TCGATCTCCAG
mWwp2 FW	CACCTACTTCCGCTTTATCGGC
mWwp2 RV	GTCGGTCTCTTGTTGAGCATCC
mHmbs FW	GAAACTCTGCTTCGCTGCATT
mHmbs RV	TGCCCATCTTTCATCACTGTATG
mActn FW	TCCACACCCGCCACCAG
mActn RV	CCTCGTCACCCACATAGGAG
mB2m FW	GGTGCTTGTCTCACTGACC
mB2m RV	CCCGTTCTTCAGCATTTGGA

Proximity ligation assay

Proximity ligation assay was performed according to the manufacturer's instructions (Merck Sigma). The Duolink PLA Mouse minus (DUO92001) and Rabbit plus (DUO92005) probes were used in combination with the Duolink In Situ Detection Reagents Far Red (DUO92013). The combinations of antibodies used for PLA on HUVECs cells are listed below:

Mouse minus	Rap1 sc-398755					
Rabbit plus	Ubiquitin ab7780	WWP2 A302-935A	Afadin 55102	Rasip1 26064	Radil 20284	Talin1 C45F1

On the mouse aorta, a different combination was used: Goat Minus probe (DUO92006) with Goat anti-Human Ubiquitin Antibody (LS BIO, LS-C348307) and Rabbit Plus probe (DUO92005) with Rabbit anti Rap1 (Cell Signaling, #2326).

***In silico* modeling**

The initial coordinates for the RASIP1 RA domain in complex with RAP1B were obtained from the Protein Data Bank (PDB: 5KHO). RAP1A and RAP1B differed in three residues (C48, E107, C139 in RAP1A; A48, D107, N139 in RAP1B) that were far from the effector binding site. Thus, we adopted the RAP1B crystal structure for the RAP1A protein constructing the RAP1A-G12V/RASIP1 complex. Residue modifications were made to the structure of the RAP1A sequence including the G12V mutation. The generated RAP1A-G12V/RASIP1 complex was further ubiquitinated (ubiquitin PDB: 1UBQ) at K31 of RAP1A-G12V. Two initial locations of ubiquitin in the ubiquitin-RAP1A-G12V /RASIP1 complexes were modeled and simulated for sampling the potential interface between RAP1A-G12V and RASIP1, which may largely cover the possible ubiquitin-binding surface of RASIP1 in the complex. In model 2, the stable and favored ubiquitin/RASIP1 interface in the complex was sampled, implying a favored ubiquitin location in the complex (Figure S2A). A similar method was applied to model the ubiquitin-RAP1A-G12V/ TLN1 complexes (RAP1B/ TLN1, PDB: 6BA6), in which ubiquitin was placed with a similar initial location as model 2 of the ubiquitin-RAP1A/RASIP1 complex. Two independent simulations were run for ubiquitin-RAP1A-G12V/TLN1 complexes.

The all-atom MD simulations were performed by using the NAMD package[5] and the CHARMM all-atom additive force field (version C36)[6]. The TIP3 water was used to solvate the systems, in which water molecules within 2.4 Å of proteins were removed to eliminate the unreasonable atom contacts. The isomeric unit cell box was set to ~110 ×110 ×110 Å^3. Na^+ and Cl^- ions were used to neutralize the system and achieve a ~0.15 mol/L ionic strength in the

water box. Covalent bonds involving hydrogen atoms were constrained. Before the production run, a series of minimization and dynamics cycles were performed for the solvents around the protein ensuring the system is fully relaxed at 310 K. Short-range van der Waals (vdW) and long-rang electrostatic interactions were individually calculated by switch function and the Particle mesh Ewald (PME) algorithm. 2 fs time-step was used in all MD simulations.

Statistics

Data entry and all analyses were performed in a blinded fashion. All statistical analyses were performed using GraphPad Prism software assuming non-parametric parameters. Statistical significance was calculated by Wilcoxon Mann-Whitney or Wilcoxon matched-pairs signed-rank on two experimental conditions. A comparison of continuous variables between more than two groups was performed by Kruskal-Wallis test and if statistical significance was observed, Dunn's Multiple Comparison Test. Two-dimensional data were analyzed by two-way ANOVA with Bonferroni post-tests. Graphs show each replicate as dots. No experiment-wide multiple test correction was applied. Proteomics and ubiquitome data were analyzed by Ingenuity pathway analysis (IPA) (QIAGEN Inc).

References

1. Sewduth, R. N. *et al.* The Noonan Syndrome Gene Lztr1 Controls Cardiovascular Function by Regulating Vesicular Trafficking. *Circ. Res.* 1379–1393 (2020)
2. Sewduth, R. N. *et al.* PDZRN3 destabilizes endothelial cell-cell junctions through a PKCζ-containing polarity complex to increase vascular permeability. *Sci. Signal.* **10**, eaag3209 (2017).
3. Chen PY, Qin L, Li G, *et al.* Endothelial TGF-β signalling drives vascular inflammation and atherosclerosis. *Nat Metab.* **1**(9):912-926 (2019).
4. Simicek, M. *et al.* The deubiquitylase USP33 discriminates between RALB functions in autophagy and innate immune response. *Nat. Cell Biol.* **15**, 1220–1230 (2013).
5. Phillips, J. C. *et al.* Scalable molecular dynamics with NAMD. *Journal of Computational Chemistry* vol. 26 1781–1802 (2005).

6. Brooks, B. R. *et al.* CHARMM: The biomolecular simulation program. *J. Comput. Chem.* **30**, 1545–1614 (2009).

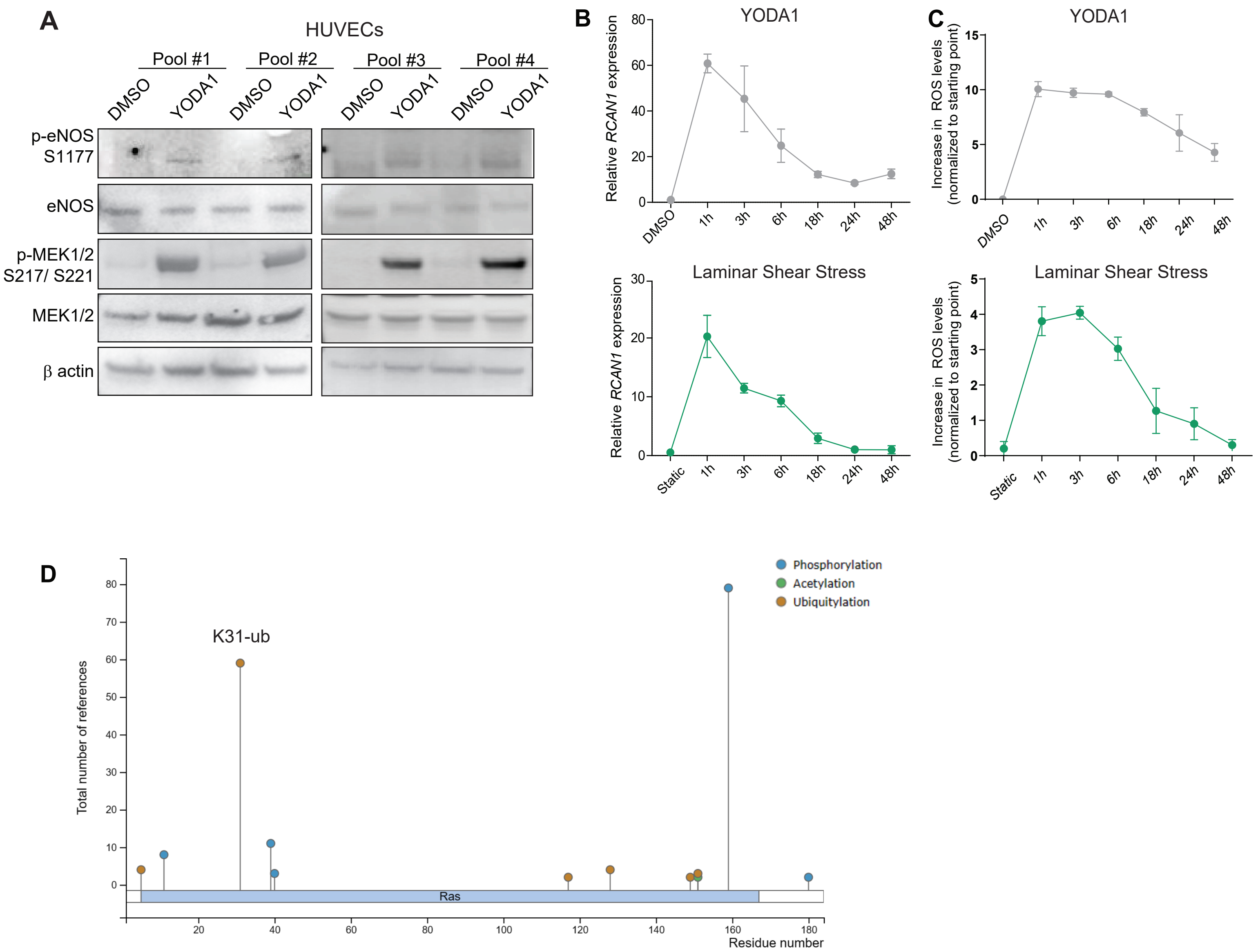

Figure S1. YODA1 treatment partially recapitulates laminar shear stress activation. (a) Four independent pools of HUVECs were treated with with 3µM YODA1 for 48 hours. Immunoblot analysis was performed using the indicated antibodies. (b) *RCAN1* expression level in YODA1- treated (3µM) or LSS (6 dyn/cm^2) HUVECs detected by RT-qPCR. Data is shown as mean ± SEM. N=3. (c) Relative cellular ROS levels measured by Cell-ROX in static, shear stress activated (0 to 48 hours; LSS=6 dyn/cm^2) or YODA1 treated (3µM) HUVECs. Data is shown as mean ± SEM. N=4 each. (d) Post-translational modifications of mouse RAP1B (adapted from Phosphosite, Cell Signaling). K31 ubiquitination is the most detected ubiquitination RAP1 site, based on 59 high-throughput mass spectrometry analysis papers.

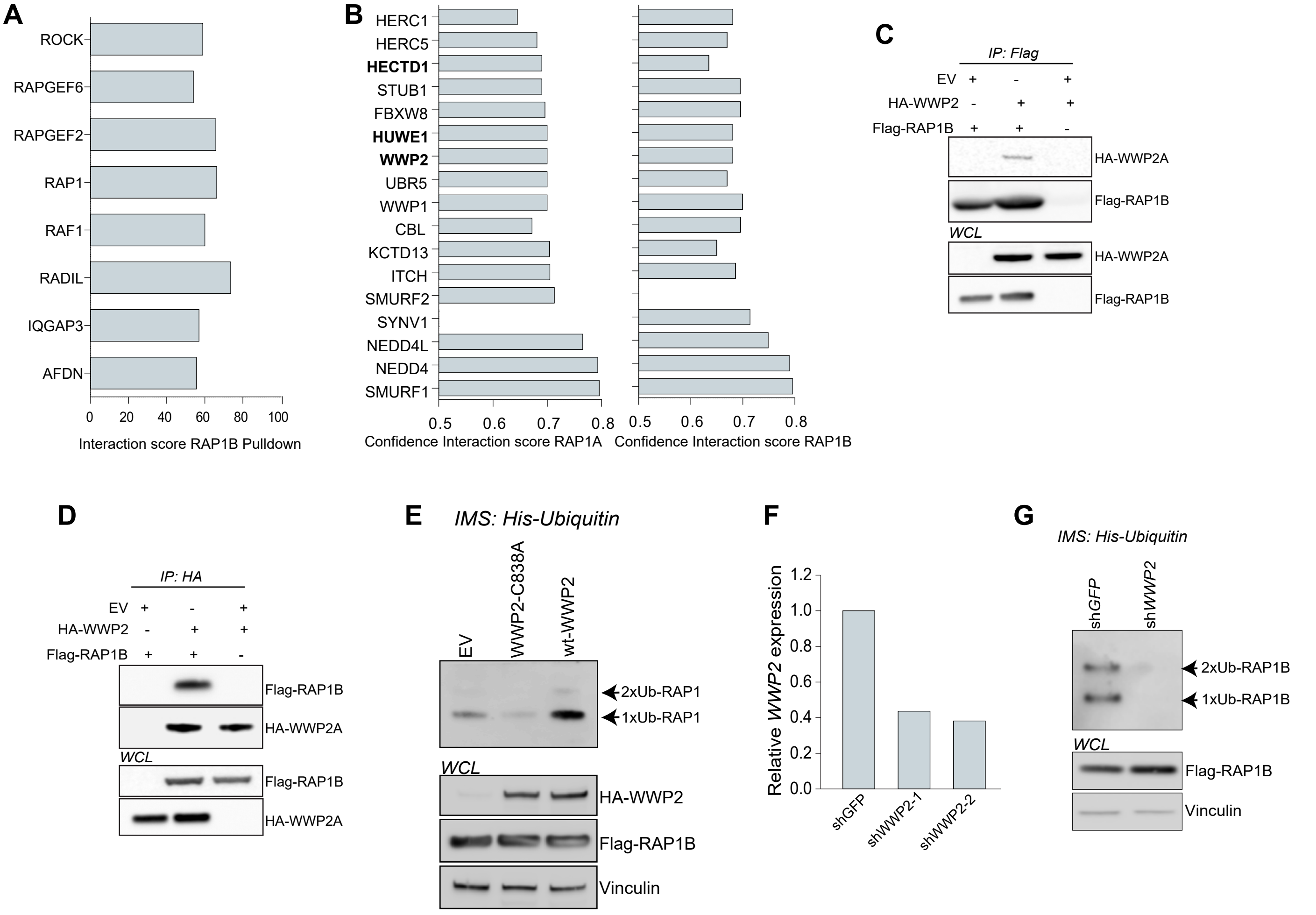

Figure S2. WWP2 controls RAP1 ubiquitination. (a) Key canonical interactors of RAP1B were identified in our MS analysis of tandem purified Flag-RAP1B overexpressed in HEK293T cells. (b) Putative interactors of RAP1A and RAP1B predictedby Ubibrowser. In orange, E3 ubiquitin ligases that scored in our MS experiment and in the Ubibrowser dataset. (c,d) After 48 h of transfection with tagged RAP1B and WWP2 expression constructs in HEK293T cells, the indicated proteins were then immuno-precipitated with anti-Flag (M2) or anti-HA–agarose, followed by immunoblotting using anti-Flag or anti-HA antibodies. (e) RAP1 ubiquitination in HEK293T expressing an empty vector (EV), HA-wt-WWP2, or HA-WWP2-C838A together with 6xHis-tagged ubiquitin and Flag–RAP1B. Ubiquitinated RAP1B was purified by Co^{2+} metal affinity chromatography and detected using anti-Flag antibodies. (f) RT-qPCR analysis of *WWP2* expression in HEK293 48 hours after trasfection with sh*GFP* or sh*WWP2*. N=1. (g) 6xHis-tagged ubiquitin and Flag–tagged RAP1 were introduced into HEK293T together with sh*GFP* or sh*WWP2*. Ubiq-uitinated RAP1B was purified by Co^{2+} metal affinity chromatography and detected by antibodies specific to Flag. WCL,whole-cell lysate; IP, immunoprecipitates; IMS: immunomagnetic separation.

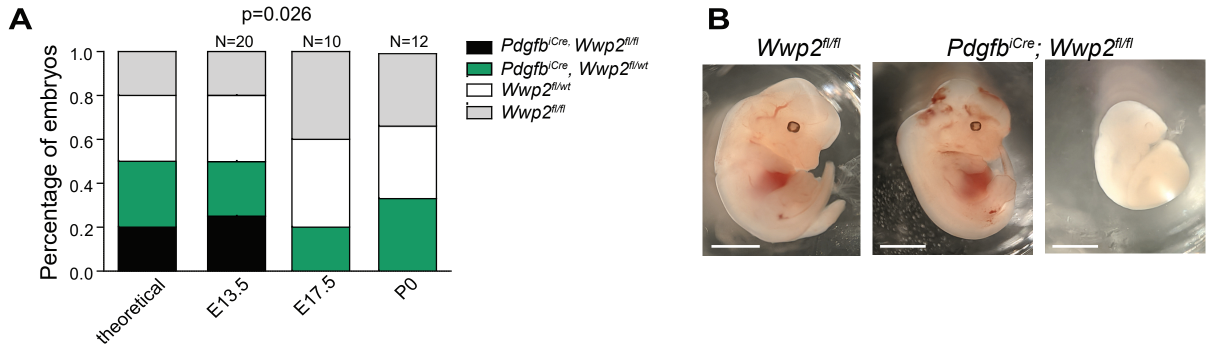

Figure S3. WWP2 is essential for RAP1 function *in vivo.* (a) Pedigree for *Pdgfb*^{iCre}*; Wwp2*$^{fl/wt}$ mice crossing. Tamoxifen was injected at E8.5, E10.5, and E12.5. p-value calculated using the One-Way ANOVA test. (b) Representative images of *Wwp2* $^{fl/fl}$ and *Pdgfb*^{iCre}*; Wwp2* $^{fl/fl}$ embryos at E13.5.Tamoxifen was injected at E8.5, E10.5 and E12.5. Scale bar, 500µm.

A

modified_sequence	start	end	score	precursor	exp.mass	cal.mass
NH2-MREYK<GGe>LVVLGSVGVGK-COOH	1	16	60	603,3346	1806,9819	1805,9822
NH2-MREYK<GGe>LVVLGSVGVGK-COOH	1	16	71	903,9967	1805,9789	1805,9822
NH2-SALTVQFVQGIFVEK<GGe>YDPTIEDSYRK-COOH	17	42	74	1049,8703	3146,5891	3146,5924
NH2-SALTVQFVQGIFVEK<GGe>YDPTIEDSYR-COOH	17	41	95	1007,1728	3018,4966	3018,4975
NH2-SALTVQFVQGIFVEK<GGe>YDPTIEDSYR-COOH	17	41	55	1007,1693	3018,4861	3018,4975

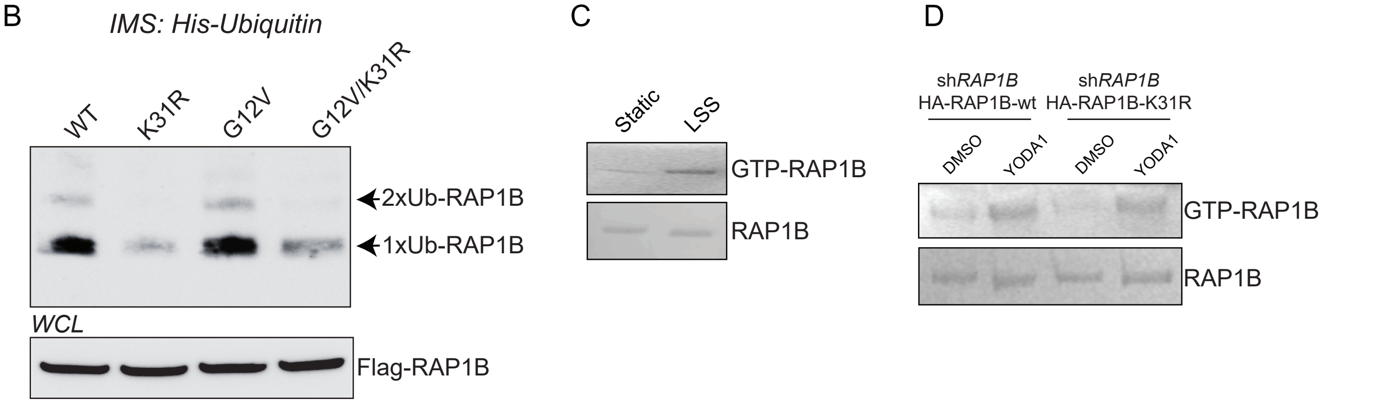

Figure S4. RAP1 activity and ubiquitination in cells exposed to shear stress and YODA1. (a) MS-based detection of RAP1B-G12V ubiquitination sites. Flag-tagged RAP1B-G12V was co-expressied with 6xHis-tagged ubiquitin and purified from HEK293T cells. (b) Ubiquitination of wt-RAP1B and the indicated RAP1B mutants. HEK-293T expressing Flag-tagged RAP1B together with 6xHis-tagged ubiquitin. Ubiquitinated RAP1B was purified by Co^{2+} metal affinity chromatography and detected using anti-Flag antibodies. (c) RAP1 activity in static or shear stress-activated HUVECs. HUVECs were subjected to LSS, 6 dyn/cm^2, 24h. (d) RAP1 activity in HUVECs expressing the indicated contructs and treated with YODA1 (3μM, 48h).

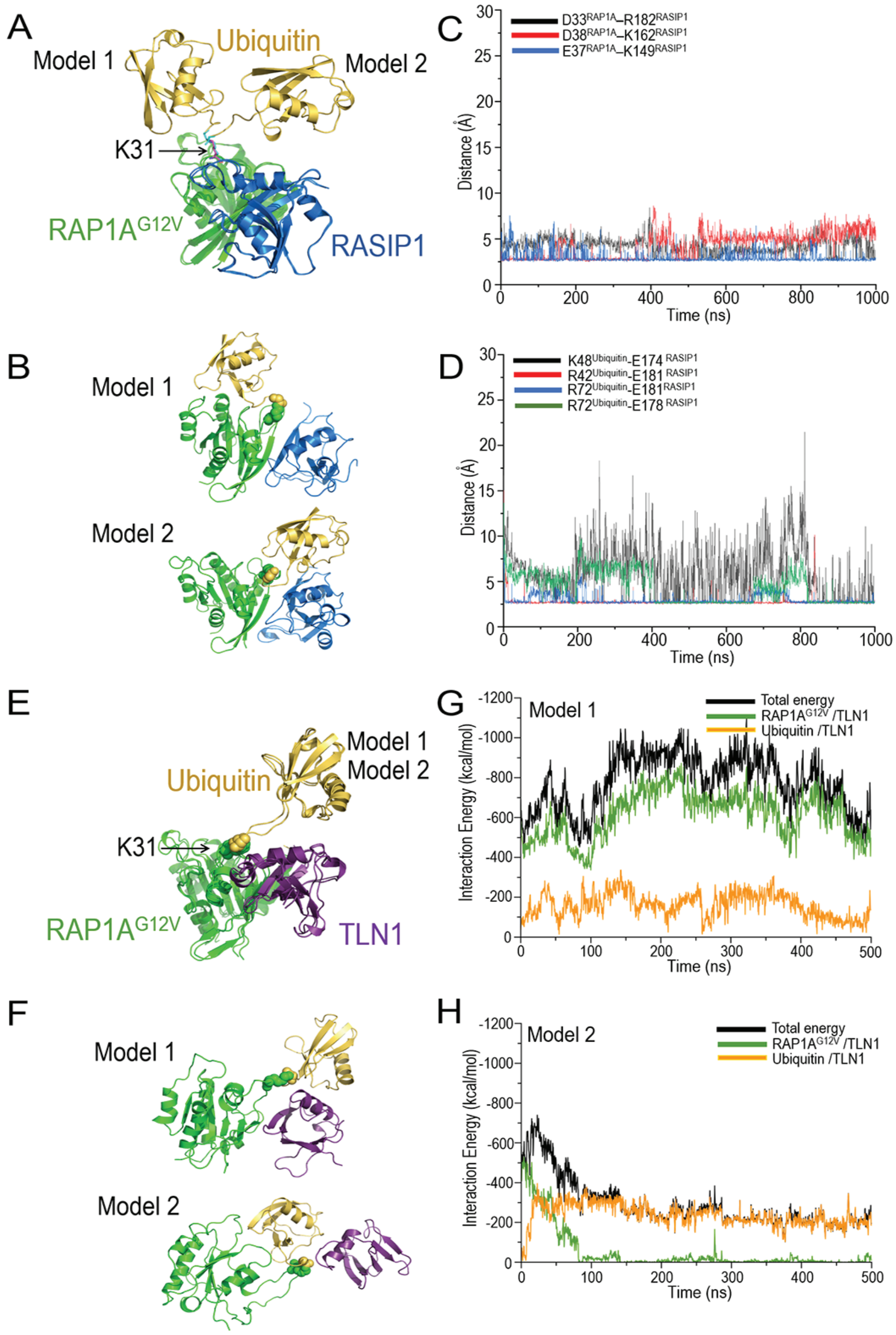

Figure S5. The interaction of ubiquitinated RAP1A with its downstream effectors. (a, b) Initial (a) and final (b) structures of the ubiquitin-RAP1A-G12V/ RASIP1 complex. (c, d) Residue contacts in the ubiquitin-RAP1A-G12V/ RASIP1 complex. (e, f) Initial (e) and final (f) structures for the ubiquitin-RAP1A-G12V/ TLN1 complex. (g,h) The interaction energies of the interaction modes in the ubiquitin-RAP1A-G12V/ TLN1 complex.

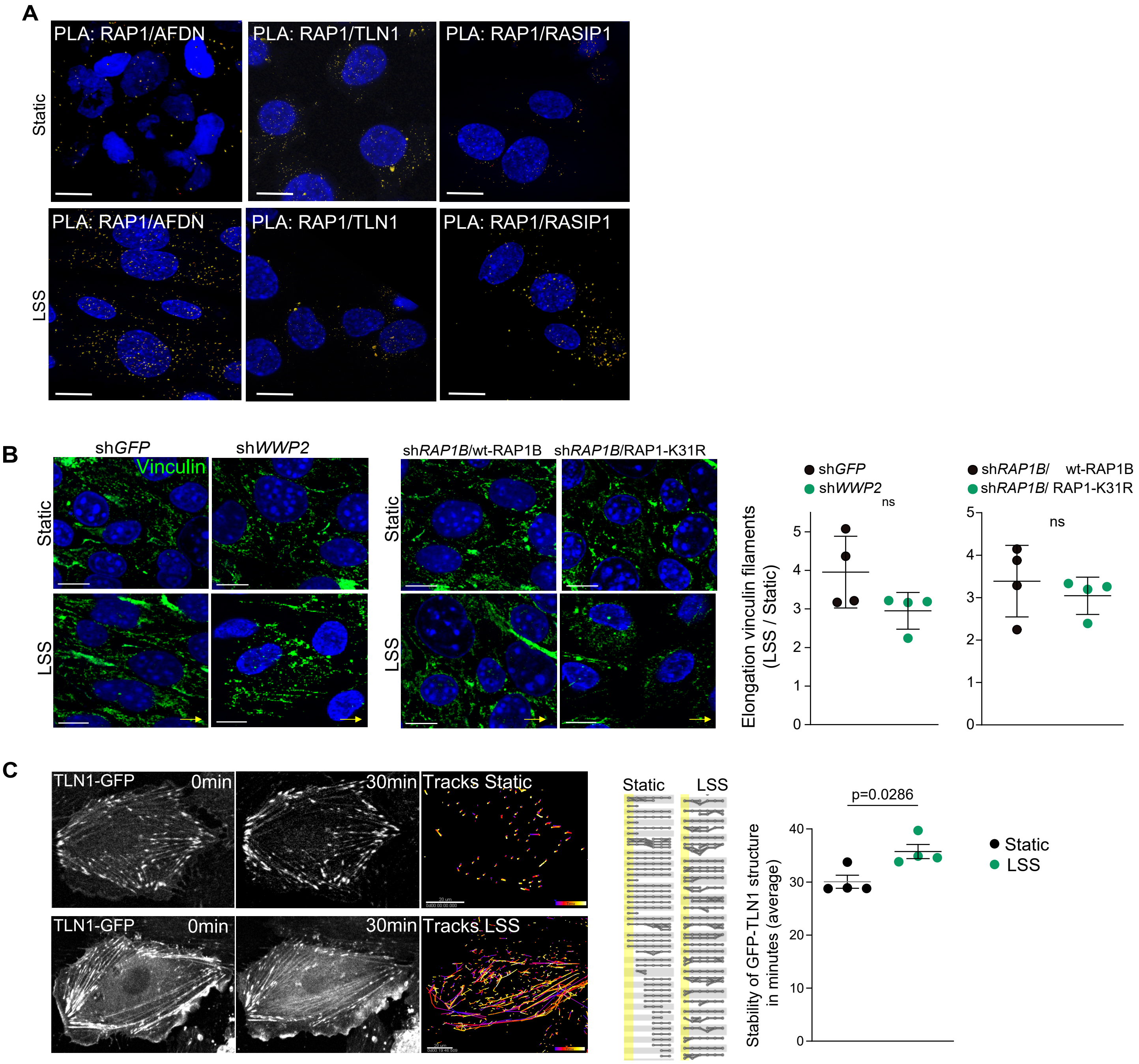

Figure S6. The interaction of RAP1 with its downstream effectors in HUVECs under shear stress. Representative images of the PLA detecting RAP1 interaction with its downstream effectors in static HUVECs or HUVECs subjected to LSS (6 dyn/cm^2). (b) Vinculin immunostaining of static or shear stress-activated HUVECs expressing the indicated constructs. HUVECs were exposed to LSS, 6 dyn/cm^2, 24h. Yellow arrows show the direction of flow. Elongation of Vinculin filaments is shown as mean ± SEM; N=4. p-values, the Wilcoxon Mann-Whitney test. (c) The stability of the TLN1 structures in HUVECs exposed to lLSS, 6 dyn/cm^2, 24h. Timelapse imaging of HUVECs transduced with TLN1-GFP BACMAM was performed for 60 minutes with acquisitions every 10min. Scale bar 5µm. The duration of the tracks during the timelapse after surfacing of the GFP-positive TLN structures is shown as mean ± SEM; N=4. p-values, the Wilcoxon Mann-Whitney test.
